# Watching the Game, Skipping the Hospital: World Cup Matches and Cardiovascular Hospitalizations

**DOI:** 10.14740/cr2271

**Published:** 2026-08-31

**Authors:** Robert Ades, Vlad Shusterman

**Affiliations:** a Department of Medicine, Donald and Barbara Zucker School of Medicine at Hofstra University, Northwell Health, Manhasset, NY, USA

**Keywords:** World Cup, Myocardial infarction, Heart failure, Healthcare utilization, Public health

## Abstract

**Background:**

Major sporting events may acutely alter patterns of cardiovascular hospitalization through changes in healthcare-seeking behavior, hospital utilization, and emotional or behavioral triggers. Brazil participated in the FIFA World Cup in 2014 (as host), 2018, and 2022, providing a unique natural experiment.

**Methods:**

We analyzed daily counts of acute myocardial infarction (AMI, ICD-10 I21) and heart failure (HF, ICD-10 I50) hospitalizations from the Brazilian national hospital information system (SIH-SUS) during three ± 30-day windows around each World Cup tournament (2014, 2018, 2022). Negative binomial regression models estimated incidence rate ratios (IRRs) for Brazil match days versus non-match days, controlling for day of week, month, and year fixed effects.

**Results:**

On Brazil match days, AMI admissions were 7.0% lower than non-match days (IRR 0.930, 95% confidence interval (CI) 0.898–0.962, P < 0.001) and HF admissions were 11.1% lower (IRR 0.889, 95% CI 0.855–0.925, P < 0.001).

**Conclusions:**

Brazil World Cup matches were associated with significant reductions in same-day AMI and HF hospitalizations. These findings highlight the potential impact of major sporting events on hospital utilization and cardiovascular care access.

## Introduction

Major international sporting events exert profound psychological and behavioral effects on entire populations. For nations with deeply emotional attachments to football, the FIFA World Cup may represent one of the most intense collective stress exposures in modern society. Two competing mechanisms may link match exposure to acute cardiovascular outcomes.

First, healthcare avoidance: during matches, people may delay or forgo seeking medical attention for symptoms that might otherwise prompt hospital presentation, an effect attributed to patients deferring care during televised broadcasts [1, 2]. Pena et al found that adult emergency department (ED) visits in Bilbao, Spain, decreased by 7.5% during local football matches and by 8.4% when matches were played away [1]. More recently, Barut & Barut reported a significant decline in ED visits during Turkish football derby matches [2]. Similar findings were reported in Lisbon, Portugal, where total ED visits decreased by 20.6% during football derby matches [3]. These studies highlight a consistent pattern across settings, whereby a share of patients who would otherwise seek care defer doing so during major sporting events.

Second, emotional stress-triggered cardiac events: intense psychological arousal, particularly from high-stakes matches and adverse outcomes such as elimination, may precipitate acute cardiovascular events through catecholamine surges, hemodynamic stress, and inflammatory pathways [4–7]. Interestingly, Carroll et al found that admissions for acute myocardial infarction (AMI) increased by 25% on the day England lost to Argentina in a penalty shoot-out and during the following 2 days [8]. Similarly, during the 2006 FIFA World Cup, Wilbert-Lampen et al reported that cardiac emergencies in Munich were 2.66 times more frequent on Germany match days compared to the same calendar dates in non-World Cup years, suggesting that emotional stress from high-stakes matches can precipitate acute cardiovascular events in the host nation [9]. Notably, Barut & Barut observed that while overall ED visits significantly declined during Turkish derby matches, the proportion of visits attributable to cardiovascular complaints increased. Barut & Barut’s findings suggest that both mechanisms, emotional-stress triggered cardiac events and healthcare avoidance, may operate simultaneously [2].

However, comparisons across studies are complicated by methodological differences. Wilbert-Lampen et al compared match days against different calendar years entirely, while studies using within-tournament comparisons, match days versus non-match days within the same seasonal window, such as Barut & Barut and Pena et al, have more consistently documented reductions in hospital presentations [1, 2, 9]. The direction of effect may therefore depend on the comparison group chosen.

Brazil offers a uniquely powerful setting in which to study these competing mechanisms. Football is a substantial part of Brazilian culture, and national team matches generate collective emotional responses of exceptional intensity [10]. Brazil participated in the FIFA World Cup as the host nation in 2014, and in Russia (2018) and Qatar (2022), providing data across three separate tournaments.

We used daily cardiovascular hospitalization data from Brazil’s national hospital information system (SIH-SUS) to estimate the association between Brazil match days and two acute cardiovascular outcomes: AMI and heart failure (HF) [11]. We hypothesized that Brazil match days would be associated with reductions in observed admissions, consistent with changes in healthcare-seeking or hospital-utilization patterns during major sporting events.

## Materials and Methods

### Data source

We used the Sistema de Informacoes Hospitalares do SUS (SIH-SUS), Brazil’s national hospital information system, which captures all inpatient admissions reimbursed by the public health system (SUS) [11]. SUS covers approximately 75% of the Brazilian population [12, 13]. Data were extracted for the years 2014, 2018, and 2022, encompassing the three World Cup tournaments in which Brazil participated during the study period. Of note, Brazil was the host nation in 2014.

Individual admission records were aggregated to daily national counts using the SIH-SUS admission date field (DT_INTER). The principal diagnosis field (DIAG_PRINC) was used to define two cardiovascular outcomes using ICD-10 codes: AMI (I21) and HF (I50). Only admissions with these codes recorded in DIAG_PRINC were included. The use of ICD-10–based case definitions for cardiovascular outcomes is consistent with prior Brazilian population-based studies using DATASUS. Several prior peer-reviewed studies have similarly identified AMI using ICD-10 code I21 and HF using I50 [14–16].

### Institutional Review Board approval

Institutional review board approval was not required because this study used publicly available, deidentified, aggregate data and did not involve identifiable private information.

### Ethical compliance

This study was conducted in accordance with applicable ethical standards for research involving human participants and with the principles of the Declaration of Helsinki.

### Exposure definition

Brazil’s match schedule was recorded for each of the three tournaments: seven matches in 2014, five matches in 2018, and five matches in 2022 (total: 17 match days). Match-day exposure was defined using the admission date field (DT_INTER, the date of hospital admission) in SIH-SUS, since the hypothesis concerns when patients sought or received care rather than when they were discharged. Brazil’s 17 matches spanned local kickoff times of 09:00–17:00 Brasilia time (UTC-3); no match began after 17:00 or risked extending past midnight local time, so each match’s exposure date corresponds unambiguously to a single SIH-SUS calendar date (Supplementary Material 1, cr.elmerpub.com).

The analysis window was defined as the 30 days before and 30 days after each tournament’s start and end dates: May 13–August 12, 2014; May 15–August 14, 2018; and October 21, 2022–January 17, 2023, providing an approximately equal-length comparison period of non-match days within the same seasonal context. Because the 2022 World Cup occurred in November–December rather than June–July, observations from 2022 were drawn from a tournament-specific comparison window spanning October 21, 2022 to January 17, 2023; the pooled models additionally adjusted for calendar month and year.

### Statistical analysis

We modeled daily admission counts using negative binomial regression. This approach is appropriate for count data, such as the number of hospital admissions per day, that exhibit more variability than a standard Poisson model assumes. Using negative binomial regression is common in healthcare utilization data. A single model was fit for each of the two cardiovascular outcomes: M1: Any Brazil match day: a binary indicator for whether Brazil played that day, controlling for day of week, month, and year fixed effects.

All models controlled for day of week (Sunday through Saturday as categorical), calendar month (to account for seasonal variation), and year fixed effects (to account for secular trends in hospitalization rates across the three tournaments). Results are reported as incidence rate ratios (IRRs) with 95% confidence intervals and two-sided P-values. We used negative binomial regression because the dispersion statistic exceeded 1, indicating that the daily admission counts varied more than expected under a Poisson model [17, 18].

All analyses were conducted in R version 4.5 using the MASS package for negative binomial regression [19].

To address the limited number of exposure days (17 total Brazil match days) and the possibility of residual autocorrelation in daily admission counts, we conducted four additional sensitivity analyses using alternative approaches to statistical inference: heteroskedasticity-robust (HC3) standard errors; autocorrelation-consistent (Newey-West) standard errors with a lag of 3 days; standard errors clustered by tournament, interpreted as exploratory given only three clusters; and a tournament-stratified permutation test. For the permutation test, match-day status was randomly reshuffled 2,000 times within each tournament, preserving the observed number of match days per tournament (seven in 2014, five in 2018, and five in 2022), and the primary negative binomial model was refit for each permutation. A two-sided permutation P-value was calculated as the proportion of permuted match-day coefficients at least as extreme in magnitude as the observed coefficient, using an add-one correction to avoid a P-value of zero.

### Statistical power

Statistical power was assessed using Monte Carlo simulation matching the primary negative binomial model specification, the observed daily admission rates, and the dispersion parameter estimated from the primary models. For each of several hypothetical match-day effect sizes, 200 datasets were simulated under the actual study design (273 observation-days, including the 17 observed match days distributed across the three tournaments), and the primary model was refit to each simulated dataset; power was defined as the proportion of simulations in which the match-day coefficient reached two-sided significance (P < 0.05). This approach indicated high power to detect the effect sizes actually observed in the primary analysis: 99.5% power for the 7.0% reduction observed for AMI and > 99% power for the 11.1% reduction observed for HF. Power remained at or above 76% for more modest reductions of 5%, and fell below 20% only for hypothetical reductions smaller than 2% (Supplementary Material 2, cr.elmerpub.com).

## Results

### Descriptive statistics

The analysis dataset comprised 273 observation-days across the three tournament windows (256 non-match days and 17 match days). Brazil played 17 matches in total: six group-stage wins, two group-stage draws, one group-stage loss, four knockout wins, and four knockout losses (including the 2014 third-place playoff). Mean daily national admissions were 347 for AMI and 595 for HF. In unadjusted descriptive comparisons, mean AMI admissions were 327 on Brazil match days versus 348 on non-match days, while mean HF admissions were 553 versus 598, respectively. These descriptive comparisons do not account for day-of-week or seasonal effects, which were subsequently adjusted for in the regression models.

### Match day effects (M1)

After adjustment for temporal confounders, Brazil match days were associated with significantly fewer AMI admissions (IRR 0.930, 95% CI 0.898–0.962, P < 0.001) and fewer HF admissions (IRR 0.889, 95% CI 0.855–0.925, P < 0.001) compared to non-match days (Table 1). Results remained significant after exclusion of the 2014 tournament, in which Brazil participated as the host nation, with significant reductions in AMI (IRR 0.947, 95% CI 0.906–0.990, P = 0.015) and HF (IRR 0.900, 95% CI 0.854–0.949, P = 0.0001) observed across the 2018 and 2022 away tournaments (Supplementary Material 3, cr.elmerpub.com).

**Table 1:** Negative Binomial Regression Results: IRRs for Cardiovascular Hospitalizations on Brazil World Cup Match Days Versus Non-Match Days, 2014–2022

Outcome	Model	Exposure	IRR	95% CI	P-value
AMI	M1: any match day	Brazil match	0.930	0.898–0.962	< 0.001
Heart failure	M1: any match day	Brazil match	0.889	0.855–0.925	< 0.001

Model controls for day of week, calendar month, and year fixed effects. Reference category for all models is non-match days within the same ± 30-day tournament window. AMI: acute myocardial infarction (ICD-10 I21); HF: heart failure (I50); IRR: incidence rate ratio.

### Exploratory match outcome analysis

In an exploratory analysis stratified by match outcome, reductions in AMI and HF hospitalizations were numerically greater on Brazil win days than on loss/draw days. However, formal comparisons between win and loss/draw days were not statistically significant for AMI (P = 0.071) or HF (P = 0.381) (Supplementary Material 4, cr.elmerpub.com).

### Post-match analysis

In post-match analyses, AMI admissions were not significantly elevated on days +1, +2, or +3 after Brazil matches. HF admissions were not significantly elevated on days +1 or +2, but showed a modest increase on day +3 (IRR 1.04, 95% CI 1.00–1.09, P = 0.044). In cumulative analyses extending the exposure window through days +1 to +3, the match-day associations for both AMI and HF progressively attenuated toward the null (Supplementary Material 5, cr.elmerpub.com).

### Robustness to alternative inference methods

The primary findings were consistent across all alternative approaches to inference (Table 2). Point estimates for the match-day effect were essentially unchanged when heteroskedasticity-robust (HC3), autocorrelation-consistent (Newey-West), and tournament-clustered standard errors were substituted for model-based standard errors, and all remained statistically significant for AMI and HF. In a tournament-stratified permutation test (2,000 permutations), no permuted match-day coefficient was as extreme as the observed coefficient for either AMI or HF (both permutation P < 0.001), providing independent, model-free support for the primary findings.

**Table 2:** Sensitivity of the Brazil Match-Day Association to Alternative Inference Methods

Outcome	Method	IRR	95% CI	P-value
AMI	Model-based SE (primary)	0.930	0.898–0.962	< 0.001
AMI	HC3 robust SE	0.930	0.894–0.967	< 0.001
AMI	Newey-West (lag 3)	0.930	0.896–0.965	< 0.001
AMI	Clustered by tournament^a^	0.930	0.893–0.968	< 0.001
AMI	Permutation (2,000 permutations)^b^	0.930	-	< 0.001
Heart failure	Model-based SE (primary)	0.889	0.855–0.925	< 0.001
Heart failure	HC3 robust SE	0.889	0.858–0.922	< 0.001
Heart failure	Newey-West (lag 3)	0.889	0.861–0.919	< 0.001
Heart failure	Clustered by tournament^a^	0.889	0.853–0.928	< 0.001
Heart failure	Permutation (2,000 permutations)^b^	0.889	-	< 0.001

^a^Clustered standard errors are exploratory given only three tournament clusters. ^b^Permutation test shuffles match-day status within tournament, preserving the observed number of match days per tournament; P-value reflects the proportion of 2,000 permuted coefficients at least as extreme as the observed coefficient (add-one correction). CI: confidence interval; IRR: incidence rate ratio; SE: standard error.

## Discussion

**Figure 1:**
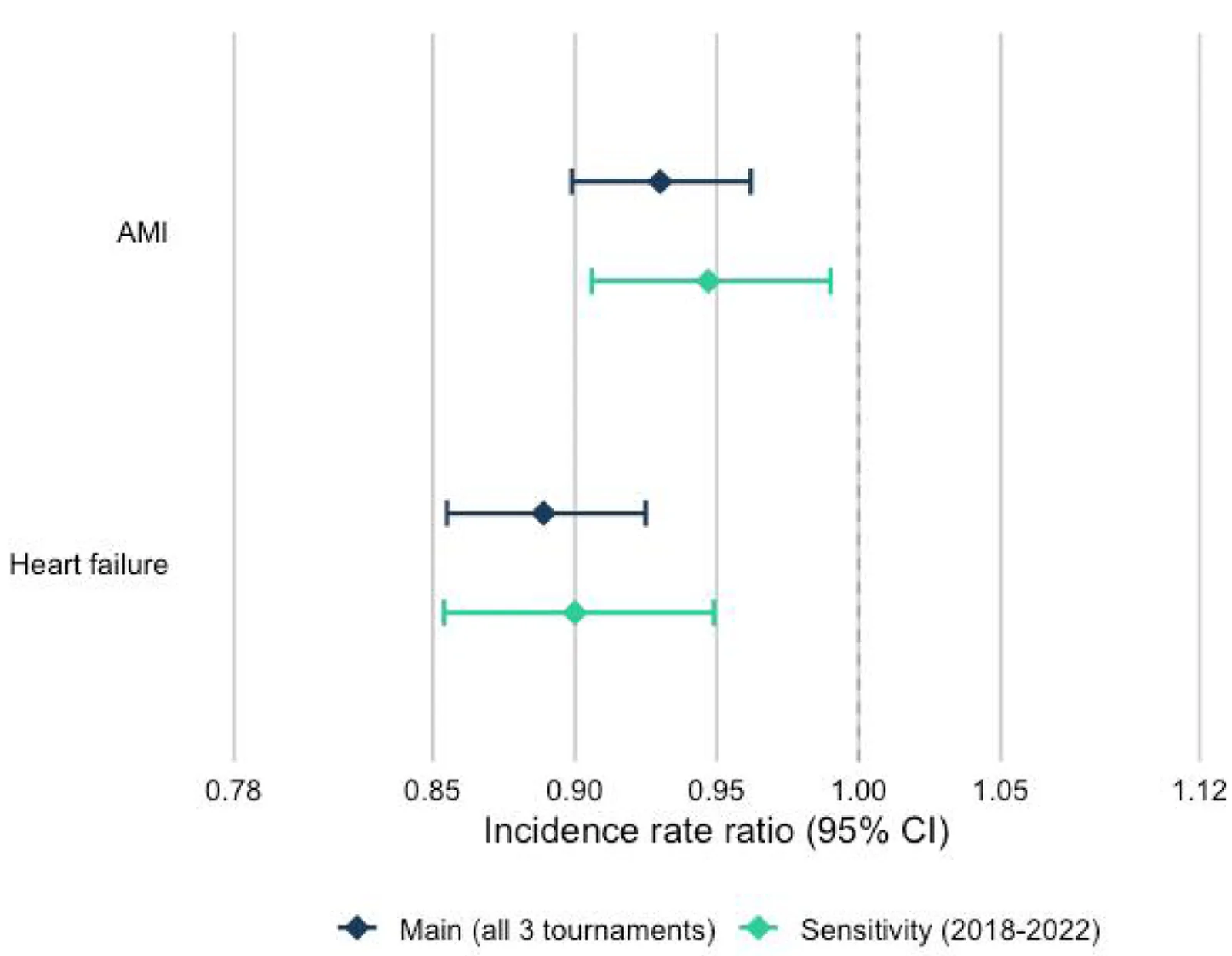
Incidence rate ratios (95% CI) for AMI and heart failure on Brazil match days versus non-match days, primary and sensitivity analyses. Blue markers show the primary analysis (all three tournaments); green markers show the sensitivity analysis excluding the 2014 host-nation tournament. AMI: acute myocardial infarction; CI: confidence interval.

This study provides evidence that Brazil’s FIFA World Cup matches are associated with significant reductions in acute cardiovascular hospitalizations on match days (Fig. 1). These findings suggest that major sporting events may alter cardiovascular hospital-utilization patterns, although the underlying mechanism cannot be determined from hospitalization data alone.

### Healthcare avoidance versus emotional stress

The observed reductions in AMI and HF admissions on Brazil match days are compatible with altered healthcare-seeking or hospital-utilization behavior, including delayed presentation, failure to present, or changes in admission practices during match broadcasts. This direction of effect is consistent with Pena et al, who reported significant decreases in ED visits during football matches in Bilbao [1]. Additionally, Almeida et al reported parallel findings in Lisbon, Portugal, demonstrating a 20.6% decrease in ED visits during Lisbon football derby matches [3]. Similar reductions in overall ED volume during major sporting events have also been described at the time of American Football matches; Antkowiak et al observed a borderline significant decrease in ED volume during games involving the New England Patriots in the United States [20].

In post-match analyses, we did not observe a consistent immediate rebound in admissions following a match. AMI admissions were not significantly elevated on days +1, +2, or +3, and HF admissions were not significantly elevated on days +1 or +2; however, HF admissions showed a modest increase on day +3 (IRR 1.04, 95% CI 1.00–1.09, P = 0.044). Examining cumulative admissions across expanding post-match windows nevertheless suggested gradual attenuation of the match-day reduction: the AMI IRR moved from 0.930 on the match day alone toward 0.980 when cumulated through day +3, and the HF IRR moved from 0.889 toward 0.980 over the same window, with both approaching the null as the window widened (Supplementary Material 5, cr.elmerpub.com). This pattern does not support a complete immediate rebound on the day following a match and may be compatible with a more diffuse normalization of hospitalization volume over the subsequent several days. However, because the analysis is based on aggregate daily hospitalization counts, it cannot determine whether these patterns reflect delayed presentation by the same patients, foregone care, changes in admission practices, or other shifts in healthcare utilization.

Whether emotional stress-triggered events occur simultaneously with healthcare avoidance is less clear. Barut & Barut observed that while overall ED visits declined during Turkish derby matches, cardiovascular complaints accounted for a larger proportion of those who did present, raising the possibility that both mechanisms may operate concurrently [2]. Unlike the reductions in AMI and HF admissions in the current study, some studies have documented absolute increases in acute cardiac events during high-stakes matches; Wilbert-Lampen et al reported cardiac emergencies in Munich were 2.66 times more frequent on Germany match days during the 2006 World Cup, and Carroll et al found AMI admissions increased by 25% on the day and the 2 days following England’s loss to Argentina on penalties [8, 9]. Together, these findings suggest that healthcare avoidance and stress-triggered events may coexist, with the dominant pattern depending on the comparison group, outcome measured, and level of care examined. Because the present analysis included only cardiovascular hospital admissions and not overall ED or hospital visit volume, we could not determine whether cardiovascular admissions represented a larger share of all presentations on match days.

Whether lower admission counts on match days were associated with worse downstream outcomes, including larger infarcts, delayed reperfusion, more severe HF decompensation, or mortality, cannot be determined from the present hospitalization data. SIH-SUS captures inpatient admissions and does not capture deaths occurring before hospital presentation or elsewhere in the community. A valid assessment of population-level cardiovascular mortality would require a separate analysis using Brazil’s mortality registry. Stress-triggered events that do occur may also present to EDs without resulting in admission and therefore would not be captured in SIH-SUS hospitalization data.

### Strengths and limitations

Strengths of this study include national coverage through SIH-SUS, objective administrative endpoints, and a quasi-experimental design using predetermined match schedules as variation in emotional exposure. The three-tournament design spanning host and away contexts enhances generalizability. Results were robust to exclusion of the 2014 host-nation tournament, with significant reductions in AMI (IRR 0.947, 95% CI 0.906–0.990, P = 0.015) and HF (IRR 0.900, 95% CI 0.854–0.949, P = 0.0001) observed across the 2018 and 2022 away tournaments (Fig. 1, Table 1). The persistence of these findings after exclusion of the 2014 host-nation tournament suggests that the observed association was not driven solely by the unique context of Brazil hosting the competition (Supplementary Material 3, cr.elmerpub.com).

Several limitations warrant consideration. First, SIH-SUS captures public-sector admissions (approximately 75% of the population), which may not fully represent privately insured patients, whose healthcare-seeking patterns during major sporting events may differ from those of SUS users [13, 21]. Second, the analysis is ecological at the national level and cannot account for regional variation in match viewership or cardiovascular risk. Third, the limited number of Brazil match days (17 across three tournaments) restricts statistical precision, particularly for very small effects and tournament-specific or heterogeneous effects. Fourth, SIH-SUS captures inpatient admissions rather than all ED encounters. We therefore could not determine whether match days affected symptom onset, emergency presentation, admission decisions, or some combination of these processes. We also could not assess out-of-hospital deaths or population-level mortality. Accordingly, the observed reductions should be interpreted as reductions in recorded cardiovascular hospitalizations, not as evidence of reduced AMI or HF incidence. Fifth, we cannot directly measure emotional arousal or stress during match broadcasts.

### Public health implications

These findings have concrete implications for public health communication during major sporting events. The observed reductions may reflect altered healthcare-seeking or hospital-utilization behavior rather than reduced disease burden. Although the absence of a consistent short-term rebound prevents us from concluding that patients simply deferred care to the following days, the results support public health messaging that encourages timely evaluation for cardiovascular symptoms regardless of match scheduling. Public health messaging during World Cup periods should therefore actively encourage individuals, particularly those with known heart failure, CAD, or prior AMI, to seek care promptly if symptoms arise and not to delay presentation because of a match.

### Conclusions

Brazil World Cup matches were associated with clinically and statistically significant reductions in same-day AMI and HF hospitalizations across three tournaments from 2014 to 2022. These associations were robust across multiple sensitivity analyses. However, the findings should be interpreted as changes in recorded hospitalizations rather than proven reductions in cardiovascular event incidence. Altered healthcare-seeking, hospital utilization, and match-day behavioral changes remain plausible explanations, but the absence of a consistent post-match rebound indicates that the underlying mechanism remains uncertain. These findings highlight the importance of distinguishing healthcare utilization patterns from underlying disease burden when interpreting administrative data during major sporting events, and support targeted public health messaging to encourage timely cardiovascular care-seeking regardless of match scheduling.

## Supplementary Material

Suppl 1 Brazil’s FIFA World Cup Matches, 2014–2022, with local kickoff times.

Suppl 2 Simulation-based statistical power by hypothetical match-day effect size.

Suppl 3 Sensitivity analysis restricted to 2018 and 2022 tournaments.

Suppl 4 Exploratory match-outcome analysis for AMI and heart failure.

Suppl 5 Individual post-match and cumulative incidence rate ratios for AMI and heart failure.

## Data Availability

The data analyzed in this study are publicly available through the Brazilian Ministry of Health Department of Informatics of the Unified Health System database, DATASUS. The analytic code and processed data used for this study are available from the corresponding author upon reasonable request.

## Abbreviations

AMI: acute myocardial infarction
CAD: coronary artery disease
CI: confidence interval
ED: emergency department
FIFA: Federation Internationale de Football Association
HF: heart failure
ICD-10: International Classification of Diseases, 10th Revision
IRR: incidence rate ratio
SIH-SUS: Sistema de Informacoes Hospitalares do Sistema Unico de Saude
SUS: Sistema Unico de Saude
