# Supplementary material for: Watching the Game, Skipping the Hospital: World Cup Matches and Cardiovascular Hospitalizations
Source: Cardiol Res. 2026 Aug 31;17(5):459–65. doi: 10.14740/cr2271 (PMC13568747; doi:10.14740/cr2271)
Supplement: Supplementary file 1 [file cr-17-05-459-s001.docx]

**Suppl 1.** Brazil’s FIFA World Cup Matches, 2014–2022, with Local Kickoff Times

| **Date** | **Opponent** | **Stage** | **Result** | **Kickoff (Brasília time)** |
| --- | --- | --- | --- | --- |
| 2014-06-12 | Croatia | Group | Win | 17:00 |
| 2014-06-17 | Mexico | Group | Draw | 16:00 |
| 2014-06-23 | Cameroon | Group | Win | 17:00 |
| 2014-06-28 | Chile | Round of 16 | Win (pens) | 13:00 |
| 2014-07-04 | Colombia | Quarterfinal | Win | 17:00 |
| 2014-07-08 | Germany | Semifinal | Loss | 17:00 |
| 2014-07-12 | Netherlands | Third-place | Loss | 17:00 |
| 2018-06-17 | Switzerland | Group | Draw | 15:00 |
| 2018-06-22 | Costa Rica | Group | Win | 09:00 |
| 2018-06-27 | Serbia | Group | Win | 15:00 |
| 2018-07-02 | Mexico | Round of 16 | Win | 11:00 |
| 2018-07-06 | Belgium | Quarterfinal | Loss | 15:00 |
| 2022-11-24 | Serbia | Group | Win | 16:00 |
| 2022-11-28 | Switzerland | Group | Win | 13:00 |
| 2022-12-02 | Cameroon | Group | Loss | 16:00 |
| 2022-12-05 | South Korea | Round of 16 | Win | 16:00 |
| 2022-12-09 | Croatia | Quarterfinal | Loss (pens) | 12:00 |

Kickoff times reflect Brasília official time (UTC−3), Brazil’s standard national reference time. All matches began and concluded within the same calendar date in Brasília time. Rows are shaded by tournament: 2014 (light blue), 2018 (light amber), 2022 (light gray).
