# Supplementary material for: Watching the Game, Skipping the Hospital: World Cup Matches and Cardiovascular Hospitalizations
Source: Cardiol Res. 2026 Aug 31;17(5):459–65. doi: 10.14740/cr2271 (PMC13568747; doi:10.14740/cr2271)
Supplement: Supplementary file 2 [file cr-17-05-459-s002.docx]

**Suppl 2.** Simulation-Based Statistical Power by Hypothetical Match-Day Effect Size

| **Outcome** | **Hypothetical Reduction** | **Target IRR** | **Power (200 simulations)** |
| --- | --- | --- | --- |
| AMI | 1% | 0.99 | 8.5% |
| AMI | 5% | 0.95 | 86.0% |
| AMI | 7.0% (observed) | 0.930 | 99.5% |
| AMI | 10% | 0.90 | 100% |
| AMI | 15% | 0.85 | 100% |
| Heart Failure | 1% | 0.99 | 11.0% |
| Heart Failure | 5% | 0.95 | 76.5% |
| Heart Failure | 10% | 0.90 | 100% |
| Heart Failure | 11.1% (observed) | 0.889 | 100% |
| Heart Failure | 15% | 0.85 | 100% |

For each hypothetical match-day incidence rate ratio (IRR), 200 datasets were simulated using the observed daily admission rates, the dispersion parameter from the primary model, and the actual study design (273 observation-days, 17 match days across three tournaments); the primary negative binomial model was refit to each simulated dataset. Power is the proportion of simulations reaching two-sided significance (p<0.05) for the match-day coefficient. “Observed” rows correspond to the effect sizes actually found in the primary analysis. IRR = incidence rate ratio.
