# Supplementary material for: Watching the Game, Skipping the Hospital: World Cup Matches and Cardiovascular Hospitalizations
Source: Cardiol Res. 2026 Aug 31;17(5):459–65. doi: 10.14740/cr2271 (PMC13568747; doi:10.14740/cr2271)
Supplement: Supplementary file 3 [file cr-17-05-459-s003.docx]

**Suppl 3.** Sensitivity Analysis Restricted to 2018 and 2022 Tournaments

| **Outcome** | **Model** | **Exposure** | **IRR** | **95% CI** | **p-value** |
| --- | --- | --- | --- | --- | --- |
| AMI | M1: Any match day (2018–2022) | Brazil match | 0.947 | 0.906–0.990 | 0.015 |
| Heart Failure | M1: Any match day (2018–2022) | Brazil match | 0.900 | 0.854–0.949 | <0.001 |

Reference category is non-match days within the same ±30-day tournament window. AMI = acute myocardial infarction (ICD-10 I21); HF = heart failure (ICD-10 I50); IRR = incidence rate ratio; CI = confidence interval.
