# Supplementary material for: Watching the Game, Skipping the Hospital: World Cup Matches and Cardiovascular Hospitalizations
Source: Cardiol Res. 2026 Aug 31;17(5):459–65. doi: 10.14740/cr2271 (PMC13568747; doi:10.14740/cr2271)
Supplement: Supplementary file 4 [file cr-17-05-459-s004.docx]

**Suppl 4.** Exploratory Match-Outcome Analysis for AMI and Heart Failure

**A. Match outcome versus non-match days**

| **Outcome** | **Exposure** | **IRR** | **95% CI** | **p-value** |
| --- | --- | --- | --- | --- |
| AMI | Win day | 0.907 | 0.868–0.947 | <0.001 |
| AMI | Loss/draw day | 0.965 | 0.916–1.020 | 0.179 |
| Heart Failure | Win day | 0.877 | 0.834–0.922 | <0.001 |
| Heart Failure | Loss/draw day | 0.908 | 0.855–0.964 | 0.002 |

**B. Formal comparison of win days versus loss/draw days**

| **Outcome** | **Exposure** | **IRR** | **95% CI** | **p-value** |
| --- | --- | --- | --- | --- |
| AMI | Win vs loss/draw | 0.940 | 0.879–1.010 | 0.071 |
| Heart Failure | Win vs loss/draw | 0.966 | 0.894–1.040 | 0.381 |

Win-day and loss/draw-day estimates in section A are relative to non-match days within the same tournament windows. Section B reports the ratio of the win-day IRR to the loss/draw-day IRR; values below 1 indicate a numerically greater reduction on win days. All negative binomial models adjust for day of week, calendar month, and year. These analyses are exploratory given the limited number of Brazil match days. AMI = acute myocardial infarction (ICD-10 I21); HF = heart failure (ICD-10 I50); IRR = incidence rate ratio; CI = confidence interval.
