# Supplementary material for: Watching the Game, Skipping the Hospital: World Cup Matches and Cardiovascular Hospitalizations
Source: Cardiol Res. 2026 Aug 31;17(5):459–65. doi: 10.14740/cr2271 (PMC13568747; doi:10.14740/cr2271)
Supplement: Supplementary file 5 [file cr-17-05-459-s005.docx]

**Suppl 5.** Individual Post-Match and Cumulative Incidence Rate Ratios for AMI and Heart Failure

| **Outcome** | **Window** | **IRR** | **95% CI** | **P-value** |
| --- | --- | --- | --- | --- |
| **A. Individual post-match days** | | | | |
| AMI | Day +1 | 1.01 | 0.978–1.05 | 0.482 |
| AMI | Day +2 | 0.998 | 0.963–1.03 | 0.898 |
| AMI | Day +3 | 1.01 | 0.973–1.04 | 0.661 |
| Heart Failure | Day +1 | 1.00 | 0.963–1.05 | 0.873 |
| Heart Failure | Day +2 | 1.01 | 0.967–1.05 | 0.717 |
| Heart Failure | Day +3 | 1.04 | 1.00–1.09 | 0.044 |
| **B. Cumulative match-day windows** | | | | |
| AMI | Match day only | 0.930 | 0.898–0.962 | <0.001 |
| AMI | Match day through +1 | 0.967 | 0.942–0.992 | 0.011 |
| AMI | Match day through +2 | 0.974 | 0.953–0.996 | 0.022 |
| AMI | Match day through +3 | 0.980 | 0.960–1.001 | 0.059 |
| Heart Failure | Match day only | 0.889 | 0.855–0.925 | <0.001 |
| Heart Failure | Match day through +1 | 0.939 | 0.911–0.968 | <0.001 |
| Heart Failure | Match day through +2 | 0.957 | 0.933–0.983 | 0.001 |
| Heart Failure | Match day through +3 | 0.980 | 0.956–1.005 | 0.121 |

Individual post-match-day models compare each specified post-match day (+1, +2, or +3) with all other observation days within the tournament windows. Cumulative models compare the indicated match-day window with observation days outside that window. All negative binomial models adjust for day of week, calendar month, and year. AMI = acute myocardial infarction (ICD-10 I21); HF = heart failure (ICD-10 I50); IRR = incidence rate ratio; CI = confidence interval.
